# Diagnostic Performance of ChatGPT‐4.0 in Histopathological Analysis of Gliomas: A Single Institution Experience

**DOI:** 10.1111/neup.70023

**Published:** 2025-07-29

**Authors:** Manuel Mazzucchelli, Serena Salzano, Rosario Caltabiano, Gaetano Magro, Francesco Certo, Giuseppe Barbagallo, Giuseppe Broggi

**Affiliations:** ^1^ Department of Medical and Surgical Sciences and Advanced Technologies “G.F. Ingrassia”, Anatomic Pathology University of Catania Catania Italy; ^2^ Department of Neurological Surgery, Policlinico “G. Rodolico‐S. Marco” University Hospital Catania Italy; ^3^ Interdisciplinary Research Center on Brain Tumors Diagnosis and Treatment University of Catania Catania Italy

**Keywords:** artificial intelligence, central nervous system tumors, ChatGPT, Gemini, neuropathology

## Abstract

This study aimed to evaluate the performance of ChatGPT‐4.0 as a diagnostic support tool for pathologists in identifying different types of gliomas based on histopathological data and to compare its performance with that of another artificial intelligence tool (Gemini 2.5 Pro). A retrospective analysis was performed on 25 cases with histopathological descriptions. The dataset, anonymized for patient confidentiality, included clinical details such as age, sex, and site, along with two histological images for each case, obtained from the archive files of the Anatomic Pathology section, Department of Medical, Surgical Sciences and Advanced Technologies “G.F. Ingrassia” University of Catania, Italy. ChatGPT‐4.0 was tasked with generating diagnoses, which were classified as correct, similar, or different when compared to the pathologists' conclusions and the diagnoses provided by Gemini. ChatGPT‐4.0 achieved a diagnostic accuracy of 88%, correctly identifying 22 out of 25 cases. No significant differences in diagnostic performance were observed between male and female patients. The AI performed exceptionally well in diagnosing glioblastomas, with a 100% accuracy rate, while two oligodendrogliomas and one astrocytoma IDH‐mutant G3 were misdiagnosed. A comparative evaluation with Gemini 2.5 Pro was also conducted, although its contribution was limited to a qualitative comparison based on the same dataset. ChatGPT‐4.0 demonstrated moderate accuracy in the histopathological diagnosis of gliomas, with little variability depending on glioma subtype. While its performance highlights potential for future integration into clinical workflows, significant improvements are required to ensure its reliability and effectiveness in diagnostic applications.

**Trial Registration:** ce 165/2015/PO

**Abbreviations**
AI: artificial intelligence
AUC: area under the curve
ChatGPT: Chat Generative Pretrained Transformer
IDH: Isocitrate dehydrogenase

## 1 Introduction

Chat Generative Pretrained Transformer (ChatGPT) is an artificial intelligence (AI) platform that leverages deep learning to answer questions in a conversational manner [1]. Developed in November 2022 by OpenAI and Microsoft, ChatGPT has shown potential in expediting clinical diagnostic reasoning, improving consultation workflows, and enhancing research systems [2]. By processing large datasets and synthesizing information, ChatGPT can assist clinicians, researchers, and healthcare professionals in tasks such as diagnostic reasoning, improving workflows, and summarizing medical literature. Although initially designed for general use, ChatGPT has demonstrated utility in various medical domains, including radiology, pathology, and clinical diagnostics. In the context of neuropathology, specifically the diagnosis of gliomas and other neurological disorders, the application of ChatGPT is an area of growing interest [3]. Neuropathology involves the complex interpretation of histopathological descriptions of brain tumors, lesions, and neurological conditions, often requiring specialized knowledge and clinical judgment [4]. With a global shortage of neuropathologists and the increasing complexity of neuropathological cases, there is a growing need for tools that can help streamline the diagnostic process. ChatGPT, with its ability to analyze large amounts of text‐based data and provide quick summaries of relevant literature, may offer significant assistance in this context. Despite its potential, ChatGPT has limitations when applied to neuropathology, particularly due to its reliance on written descriptions rather than the analysis of histological images. While machine learning models in neuropathology often focus on image‐based recognition, ChatGPT's strength lies in processing and interpreting text. This distinction is crucial, as the AI's performance depends heavily on the clarity, accuracy, and quality of the written reports it analyzes. Additionally, ChatGPT lacks the reasoning, clinical judgment, and personal expertise that trained neuropathologists bring to the diagnostic process [4]. As such, while it can support pathologists by referencing current literature and guidelines, human oversight remains essential. This study evaluates ChatGPT‐4.'s diagnostic performance in interpreting histopathological descriptions of gliomas. By comparing its accuracy with that of trained neuropathologists, the study aims to assess the potential role of ChatGPT in assisting the diagnostic process in neuropathology. Through this investigation, the present study seeks to explore how AI tools like ChatGPT can complement human expertise, ultimately enhancing the efficiency and effectiveness of healthcare delivery in the field of neuropathology. In this study, we compared the performance of ChatGPT‐4.0 with another large language model (Gemini 2.5 Pro) to explore their potential as support tools in neuropathological diagnosis. Recent evidence supports the potential role of large language models as diagnostic support systems in neuropathology, rather than standalone diagnostic tools. In line with this, the current study assesses how ChatGPT‐4.0 may assist pathologists in interpreting histopathological descriptions of gliomas [5].

## 2 Methods

### 2.1 Data Collection

A total of 25 histopathological descriptions of different types of gliomas (*n* = 25) were retrospectively retrieved from the Anatomic Pathology section, Department of Medical, Surgical Sciences and Advanced Technologies “G.F. Ingrassia” University of Catania, Italy. This retrospective study was conducted in accordance with the ethical standards of the institutional and national research committees and with the 1964 Helsinki Declaration and its later amendments. Ethical approval was obtained from the Local Ethics Committee of the University of Catania, Catania 1 (ce 165/2015/PO). Given the retrospective nature of the study and the use of fully anonymized data, the requirement for informed consent was waived by the Ethics Committee. The cases of the study were recorded from February 2022 to December 2024. The anonymized data was compiled into a Microsoft Excel spreadsheet. All histopathological descriptions were originally written by board‐certified pathologists, and the cases included were those available during the specified time period that matched the inclusion criteria (typical morphological aspects and expression of the typical immunohistochemical markers for each type of glioma, mutational profile, two clear histological images for each case).

The patients included in our series were 17 females and 8 males, with a mean age of 61.7 years (range 33–77 years). The tumors were all located in various cerebral lobes, except for one case which was located in the cerebellum. The pathologists' diagnoses were Glioblastoma, Isocitrate dehydrogenase (IDH)‐wild type (15 cases); Oligodendroglioma, IDH‐mutant (6 cases, of which 4 were Grade 3 and 2 was Grade 2); Astrocytoma, IDH‐mutant (4 cases, both Grade 3). Information on the age, sex, and site of each case was included in the analysis. To ensure confidentiality, data were anonymized and numerically coded, starting from number 1, as they would be presented to pathologists in clinical practice.

### 2.2 Data Input and Use of ChatGPT

**FIGURE 1:**
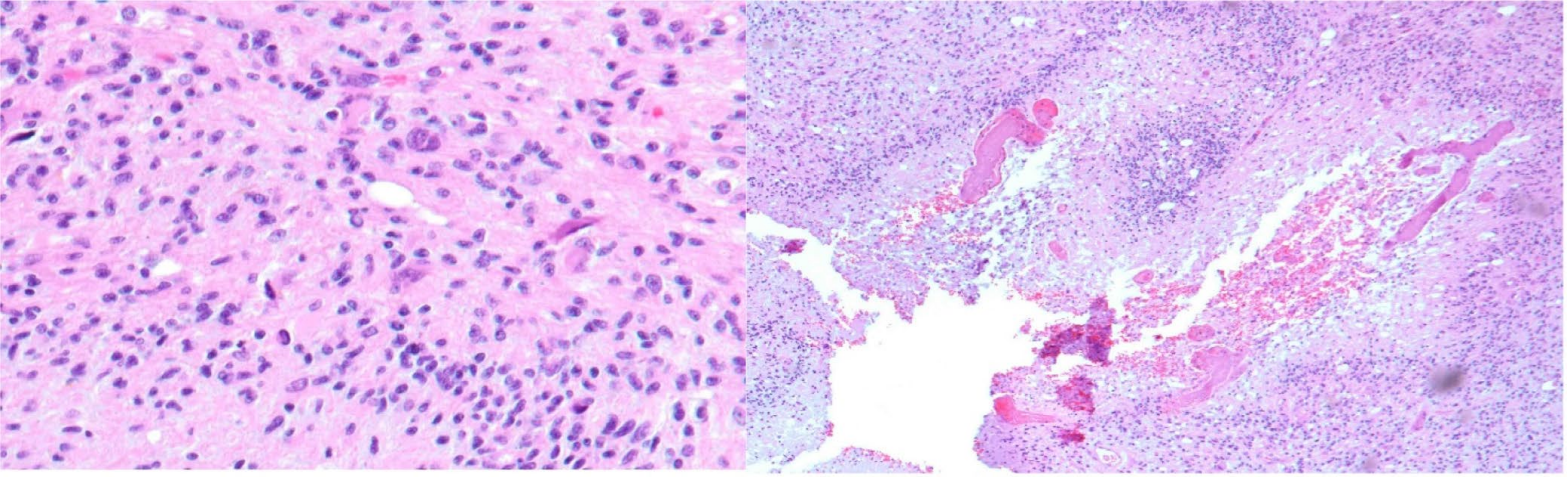
Example of photos submitted to ChatGPT (Glioblastoma, IDH‐ wild type).

Using the premium version of ChatGPT‐4.0 (OpenAI, Microsoft Corporation, version GPT‐4‐0125‐preview), the following data were recorded for each case: age, sex, localization, and histopathological description. Table 1 summarizes the representative prompt used and how text data were presented to the model. ChatGPT was provided with the same details available to the pathologists, excluding any patient identifiers, to ensure an unbiased comparison. At first, only these data were provided to process the diagnosis, without histological images. Subsequently, for each case, two histological images of representative tumor areas were provided (Figure 1). These images were carefully selected by two pathologists from the University of Catania, highlighting the typical morphological features according to the type of glioma (e.g., areas of necrosis and vascular proliferation for glioblastomas). The dataset concluded with the question: “What is the diagnosis?”. This standardization guaranteed consistency in the information provided to ChatGPT, replicating the clinical setting in which pathologists would typically receive such details. In this way, it was also possible to evaluate whether the histological images were important or not for the diagnostic purposes. Importantly, for all cases the histopathological descriptions included the immunohistochemical IDH1 (R132H) status (negative, wild‐type; positive, mutant) and 1p/19q codeletion status (present or absent). These details were also incorporated into the chatbot's input. A second output for each case was made after a week and finally a third output after a month. The results were overlapping.

**TABLE 1:** Data input for ChatGPT.

Age	Gender	Localization	Histopathological description	Pathologist's diagnosis	Chat GPT's diagnosis	Score
77	Female	Temporal	Glial tumor which was IDH‐wild type on immunohistochemistry.	Glioblastoma, IDH‐wild type	Glioblastoma, IDH‐wild type	3
66	Female	Parietal	Glial tumor with areas of necrosis and microvascular proliferation, and immunohistochemically it was IDH‐wild type.	Glioblastoma, IDH‐wild type	Glioblastoma, IDH‐wild type	3
74	Female	Insula	Glial tumor which was IDH‐wild type on immunohistochemistry.	Glioblastoma, IDH‐wild type	Glioblastoma, IDH‐wild type	3
66	Female	Cerebellar	Glial tumor with areas of necrosis and microvascular proliferation, and immunohistochemically it was IDH‐wild type.	Glioblastoma, IDH‐wild type	Glioblastoma, IDH‐wild type	3
74	Male	Parietal	Glial tumor which was IDH‐wild type on immunohistochemistry.	Glioblastoma, IDH‐wild type	Glioblastoma, IDH‐wild type	3
38	Female	Temporal	Glial tumor with hypercellularity, moderate atypia, scattered mitotic activity and areas with gemistocytic differentiation; neither necrosis nor microvascular proliferation were found. Immunohistochemical analysis showed that the tumor was IDH‐mutant.	Astrocytoma, IDH‐mutant (G3)	Astrocytoma, IDH‐mutant (G3)	3
63	Female	Frontal	Glial tumor with hypercellularity, mitotic activity up to 7 mitoses/10 HPFs and foci of microvascular proliferation. Immunohistochemical analysis showed that the tumor was IDH‐mutant. 1p/19q codeletion present.	Oligodendroglioma, IDH‐mutant and 1p/19q codeleted (G3)	Oligodendroglioma, IDH‐mutant and 1p/19q codeleted (G3)	3
64	Female	Parietal/occipital	Glial tumor which was IDH‐wild type on immunohistochemistry.	Glioblastoma, IDH‐wild type	Glioblastoma, IDH‐wild type	3
42	Male	Temporal/paretal/occipital	Glial tumor with areas of necrosis and microvascular proliferation, and immunohistochemically it was IDH‐wild type.	Glioblastoma, IDH‐wild type	Glioblastoma, IDH‐wild type	3
76	Female	Frontal	Glial tumor with foci of microvascular proliferation, and immunohistochemically it was IDH‐wild type.	Glioblastoma, IDH‐wild type	Glioblastoma, IDH‐wild type	3
67	Male	Temporal/occipital	Glial tumor with areas of necrosis and microvascular proliferation, and immunohistochemically it was IDH‐wild type.	Glioblastoma, IDH‐wild type	Glioblastoma, IDH‐wild type	3
59	Male	Parietal	Diffuse glial tumor with anaplastic features and up to 6 mitoses/10 HPF; neither necrosis nor microvascular proliferation were found. Immunohistochemistry showed that the tumor was IDH‐mutant. 1p/19q codeletion present.	Oligodendroglioma, IDH‐mutant and 1p/19q codeleted (G3)	Pilocytic astrocytoma (G1)	1
54	Female	Frontal	Diffuse glial tumor with anaplastic features and up to 13 mitoses/10 HPF; necrotic areas and microvascular proliferation were present. Immunohistochemistry showed that the tumor was IDH‐mutant. 1p/19q codeletion present.	Oligodendroglioma, IDH‐mutant and 1p/19q codeleted (G3)	Oligodendroglioma, IDH‐mutant and 1p/19q codeleted (G3)	3
65	Female	Frontal	Glial tumor with areas of necrosis and microvascular proliferation, and immunohistochemically it was IDH‐wild type.	Glioblastoma, IDH‐wild type	Glioblastoma, IDH‐wild type	3
67	Female	Not available	Glial tumor with no nuclear atipia, low mitotic index (< 1 mitosis/10 HPF); neither necrosis nor microvascular proliferation were found. Immunohistochemistry showed that the tumor was IDH‐mutant. 1p/19q codeletion present.	Oligodendroglioma IDH‐mutant and 1p/19q codeleted (G2)	Oligodendroglioma, IDH‐mutant and 1p/19q codeleted (G2)	3
49	Male	Parietal	Diffuse glial tumor without evidence of necrosis and microvascular proliferation. Immunohistochemistry showed that the tumor was IDH‐mutant.	Astrocytoma, IDH‐mutant (G3)	Diffuse Astrocytoma, IDH‐mutant (G2)	2
69	Female	Multifocal	Glial tumor which was IDH‐wild type on immunohistochemistry.	Glioblastoma, IDH‐ wild type	Glioblastoma, IDH‐ wild type	3
74	Female	Intraventricular	Glial tumor with areas of necrosis and microvascular proliferation, and immunohistochemically it was IDH‐wild type.	Glioblastoma, IDH‐ wild type	Glioblastoma, IDH‐ wild type	3
73	Female	Fronto‐parietal	Glial tumor with areas of necrosis and microvascular proliferation, and immunohistochemically it was IDH‐wild type.	Glioblastoma, IDH‐ wild type	Glioblastoma, IDH‐ wild type	3
76	Male	Fronto‐temporal	Glial tumor which was IDH‐wild type on immunohistochemistry.	Glioblastoma, IDH‐ wild type	Glioblastoma, IDH‐ wild type	3
66	Female	Fronto‐nuclear	Glial tumor with areas of necrosis and microvascular proliferation, and immunohistochemically it was IDH‐wild type.	Glioblastoma, IDH‐ wild type	Glioblastoma, IDH‐ wild type	3
51	Male	Temporal	Diffuse glial tumor without evidence of necrosis and microvascular proliferation. Immunohistochemistry showed that the tumor was IDH‐mutant.	Astrocitoma, IDH‐mutato (G2)	Diffuse astrocytoma, IDH‐mutant (G2)	3
40	Female	Parietal	Glial tumor with no nuclear atipia, low mitotic index (< 1 mitosis/10 HPF); neither necrosis nor microvascular proliferation were found. Immunohistochemistry showed that the tumor was IDH‐mutant. 1p/19q codeletion present.	Oligodendroglioma, IDH‐mutant and 1p/19q codeleted (G2)	Pilocytic astrocytoma (G1)	1
60	Female	Frontal	Diffuse glial tumor, with 6 mitoses/10 HPF and foci of microvascular proliferation. Immunohistochemistry showed that the tumor was IDH‐mutant. 1p/19q codeletion present.	Oligodendroglioma, IDH‐mutant and 1p/19q codeleted (G3)	Oligodendroglioma, IDH‐mutant and 1p/19q codeleted (G3)	3
33	Male	Not available	Glial tumor with no nuclear atipia, low mitotic index (< 1 mitosis/10 HPF); neither necrosis nor microvascular proliferation were found. Immunohistochemistry showed that the tumor was IDH‐mutant.	Astrocitoma, IDH‐mutato (G2)	Diffuse astrocytoma, IDH‐mutant (G2)	3

Furthermore, the exact same process, with the same case descriptions and images from the study, was proposed to another model (Gemini 2.5 Pro) to compare the specific skills and any differences between the two models.

### 2.3 Diagnostic Coding and Pathologist Consensus

The diagnoses generated by ChatGPT‐4.0 were subsequently compared to the original diagnoses made by pathologists and classified into the following categories, using a scoring system ranging from 1 to 3:

- Score 1: ChatGPT's diagnosis was clinically or pathologically distinct from the original diagnosis provided by the pathologists (Different).
- Score 2: Correct diagnosis but with some discrepancies, such as grading (Similar).
- Score 3: ChatGPT's diagnosis was identical to the original diagnosis made by the pathologists (Correct).

To ensure consistency and minimize subjectivity in the categorization process, a panel of two board‐certified pathologists reviewed the original histopathological descriptions. This review aimed to confirm diagnostic accuracy and consistency. The panel reached a consensus on each description and diagnosis, establishing a reliable reference standard for evaluating ChatGPT's diagnostic performance. This approach reduced variability in the reference diagnoses and enhanced the reliability of the study's findings.

## 3 Results

### 3.1 Descriptive Statistics

Comparing the results of the ChatGPT diagnoses with histological images and without histological images, this study was able to consider images as a crucial turning point for the diagnosis. In fact, in most cases without histological images, ChatGPT failed to arrive at a definitive diagnosis, but only provided us with possible differential diagnoses. Therefore, this study will only take into account the results obtained from the cases described together with the relative images.

**FIGURE 2:**
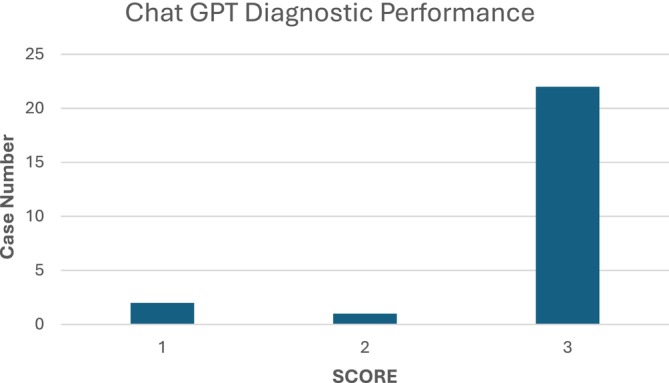
Chat GPT diagnostic performance.

ChatGPT‐4.'s diagnostic performance was grouped into three categories, compared with the pathologists' diagnosis: Different (score = 1), Similar (score = 2), and Correct (score = 3). The distribution of cases across these categories was as follows: 22 diagnoses were classified as Correct, 2 as Similar, and 1 as Different (Figure 2).

To assess the overall accuracy of ChatGPT's diagnoses, the proportion of correct diagnoses (score = 3) was calculated relative to the total number of diagnoses. Out of 25 cases, 22 were correct, yielding an overall accuracy of approximately 88%.

Regarding Gemini, however, the diagnoses remained consistent both with the support of histological images and based only on histopathological description. Furthermore, all diagnoses were concordant with ChatGPT's, except for one oligodendroglioma case which Gemini diagnosed as glioblastoma and ChatGPT as pilocytic astrocytoma. This suggests that the Gemini version has developed enhanced capabilities in predicting complex diagnoses such as those of gliomas, even in the absence of histological images, whereas ChatGPT has not yet achieved this ability. Although Gemini produced consistent diagnoses across cases, the comparison remained qualitative and was based on a limited number of outputs. Thus, a quantitative evaluation of Gemini's diagnostic performance was beyond the scope of this study.

### 3.2 Gender Influence

**FIGURE 3:**
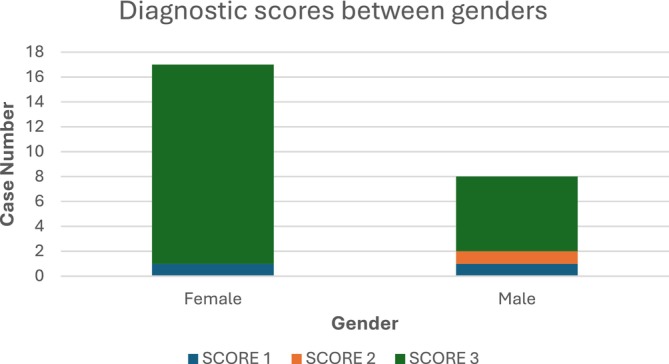
Diagnostic scores between genders.

No statistically significant difference in diagnostic scores between genders was found (Figure 3). In detail, for female patients, a score of 3 was assigned in 16 cases, whereas for male patients, a score of 3 was assigned in 6 cases, a score of 2 in 1 case, and a score of 1 in the remaining case.

### 3.3 Performance by Lesion Type

ChatGPT‐4.'s performance varied considerably depending on the type of lesion. The best results were seen with glioblastoma, IDH‐wild type cases, where 100% of the diagnoses were correct (score = 3).

However, despite the known information on the presence of 1p/19q codeletion, two oligodendroglioma cases were misdiagnosed by ChatGPT as pilocytic astrocytomas (G1).

**FIGURE 4:**
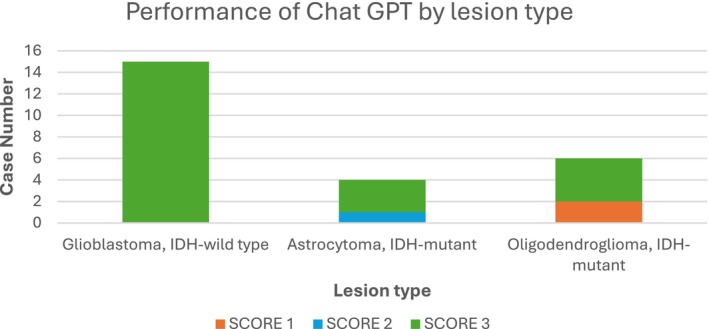
Performance of Chat GPT by type of gliomas.

Three out of four cases of Grade 3 Astrocytoma, IDH‐mutant were correctly diagnosed, while in the other case ChatGPT made a diagnosis of Diffuse Astrocytoma, IDH‐mutant (grade 2) (Figure 4).

## 4 Discussion

The AI into healthcare, particularly in specialized fields such as neuropathology, represents a rapidly evolving area of research with significant implications for clinical practice [6]. This study sought to evaluate the performance of ChatGPT‐4.0 in diagnosing gliomas based on histopathological descriptions and images, comparing its diagnoses with those of expert pathologists. The analysis provided valuable insights into both the strengths and limitations of AI chatbots within this highly specialized domain. ChatGPT‐4.0 achieved an overall diagnostic accuracy of 88%. Among the analyzed cases, it rendered the correct diagnosis in 22 cases, 2 cases were classified as similar to the pathologists' diagnosis, and provided divergent diagnoses in 1 case. These findings illustrate that while ChatGPT‐4.0 demonstrates a considerable capacity for accurate diagnosis in many scenarios, there remains a notable gap when compared to the precision required in clinical practice. This aligns with broader observations in the literature, where AI tools have shown promising results but often require integration with human expertise to achieve reliable outcomes [7].

The study also highlighted the impact of language and terminology on AI performance. ChatGPT‐4.0's diagnostic accuracy was significantly affected when histopathological descriptions deviated from standard linguistic patterns. Variability in language, a hallmark of medical reporting, poses challenges for AI, which relies heavily on consistency in input data. This suggests that achieving optimal utility for AI in diagnostics may require the adoption of standardized terminology, a recommendation echoed by several studies [8, 9]. Interestingly, the AI's performance mirrored findings from previous research. Bejnordi et al., for example, found that AI systems excel in recognizing patterns and categorizing structured data but struggle with nuanced, complex diagnostic tasks requiring contextual knowledge. In neuropathology, where subtle morphological variations can have profound diagnostic implications, this limitation becomes particularly pronounced [6]. Although a possible trend of lower diagnostic accuracy was noted in older patients, the limited sample size precluded a formal statistical analysis of this association, indicating that ChatGPT‐4.0's performance diminishes with increasing patient age [10]. This decline may stem from the complexity of age‐related histopathological changes, which require more sophisticated interpretation. Conversely, no significant differences were noted in diagnostic accuracy between male and female patients, consistent with prior studies suggesting that demographic factors may influence AI performance only in certain contexts [11].

ChatGPT‐4.0's diagnostic accuracy also varied across glioma subtypes. The AI performed exceptionally well with glioblastomas, achieving a perfect 100% accuracy rate. Glioblastomas are characterized by distinct and consistent histopathological features, which likely contributed to the high success rate. However, the AI struggled with oligodendrogliomas, failing to provide correct diagnoses in these cases. Oligodendrogliomas often present more complex and variable histological patterns, underscoring the importance of domain‐specific expertise in their interpretation. This disparity in performance emphasizes the need for training AI systems on more diverse datasets encompassing a broad spectrum of pathological presentations [6, 12].

In general pathology, AI systems have demonstrated considerable variability in diagnostic accuracy based on the complexity of the task. For instance, Campanella et al. [13] reported an area under the curve (AUC) of 0.966 for deep learning algorithms in detecting metastatic breast cancer in lymph node biopsies, a task defined by relatively clear pathological markers. Similarly, Bejnordi et al. [10] demonstrated that AI models could match expert pathologists in detecting breast cancer metastases in sentinel lymph nodes. Other studies have shown good performance of AI systems in other fields, e.g., the diagnosis of prostate cancer [14] and of head and neck cancer [15]. These findings suggest that AI excels in tasks with well‐defined pathological features and ample training data [16]. However, the lower accuracy observed in this study (88% for ChatGPT‐4.0 in neuropathology) highlights the challenges of more intricate diagnostic scenarios, where subtle morphological differences and interpretive nuances play a critical role.

These results underline the importance of adapting AI systems to specific diagnostic contexts. Enhanced algorithms capable of interpreting more complex and variable pathological presentations are essential. Future research should focus on refining AI's ability to navigate such complexities and on integrating diverse datasets that combine text‐based and image‐based inputs. Hybrid models that leverage the combined strengths of AI and expert pathologists could offer the most promising path forward, balancing efficiency with accuracy. The potential for a symbiotic relationship between human clinicians and AI has been noted by Lee, who emphasized that combining the predictive capabilities of AI with human expertise could significantly improve patient outcomes [17]. While tools like ChatGPT‐4.0 are not yet suitable for standalone diagnostic use in neuropathology, they hold considerable promise as adjuncts to human decision‐making processes.

This study also shed light on key limitations. The histopathological descriptions and images used for diagnosis were generated by the same individuals who provided the final diagnoses. This could introduce bias, as the descriptions may inherently reflect the diagnostic conclusions. Despite this, the results remain relevant for understanding the potential and limitations of conversational AI in this field. Another limitation is the sample size; a larger dataset incorporating cases from multiple institutions would provide more generalizable insights. ChatGPT‐4.0 was selected for this study due to its accessibility, advanced natural language processing capabilities, and widespread application in various sectors, including healthcare. While other AI platforms are available, ChatGPT‐4.0 serves as a representative example of conversational AI's current state of development. However, the findings of this study are specific to this model and may not be directly applicable to other AI tools.

While ChatGPT‐4.0 shows promising potential in the field of neuropathology, particularly in supporting clinical workflows and offering preliminary diagnostic insights, its performance remains inconsistent when it comes to histopathological descriptions of gliomas. This variability highlights a notable limitation in its current diagnostic capabilities, especially in distinguishing between complex glioma subtypes. While Gemini appeared more consistent in a specific case, the limited nature of the comparison prevents us from drawing strong conclusions regarding its overall superiority. The Gemini model demonstrated a more robust and consistent performance, maintaining diagnostic accuracy regardless of whether histological images were provided. Notably, all of Gemini's diagnoses aligned with those of ChatGPT, except in one case: an oligodendroglioma which Gemini identified as glioblastoma, whereas ChatGPT classified it as pilocytic astrocytoma. This discrepancy underscores ChatGPT's current challenges in accurately diagnosing certain glioma types and suggests that, given the minimal number of discrepancies and the qualitative nature of the comparison, further studies are needed before drawing definitive conclusions about the relative performance of Gemini versus ChatGPT. These findings emphasize the need for further research and refinement to enhance ChatGPT's reliability in complex neuropathological assessments.

In conclusion, this study provides a starting point for evaluating the role of conversational AI in histopathological diagnostics, and in particular in the field of neuropathology. While ChatGPT‐4.0 demonstrates significant potential, particularly in providing preliminary diagnoses and supporting clinical workflows, its limitations must be addressed through continued research, standardization, and collaboration between AI developers and medical professionals.

## Ethics Statement

Ethical approval of the research protocol was obtained from the Local Ethics Committee of the University of Catania, Catania 1.

## Consent

Given the retrospective nature of the study and the use of fully anonymized data, the requirement for informed consent was waived by the Local Ethics Committee.

## Conflicts of Interest

The authors declare no conflicts of interest.

## Data Availability

The data that support the findings of this study are available from the corresponding author upon reasonable request.

## References

[1] S. Stoneham , A. Livesey , H. Cooper , and C. Mitchell , “ChatGPT Versus Clinician: Challenging the Diagnostic Capabilities of Artificial Intelligence in Dermatology,” Clinical and Experimental Dermatology 49 (2023): llad402, 10.1093/ced/llad402. 37979201

[2] V. Sorin , E. Klang , M. Sklair‐Levy , et al., “Large Language Model (ChatGPT) as a Support Tool for Breast Tumor Board,” Npj Breast Cancer 9, no. 1 (2023): 44, 10.1038/s41523-023-00557-8. 37253791 PMC10229606

[3] G. Cazzato , M. Capuzzolo , P. Parente , et al., “Chat GPT in Diagnostic Human Pathology: Will It Be Useful to Pathologists? A Preliminary Review With “Query Session” and Future Perspectives,” Ai 4 (2023): 1010–1022, 10.3390/ai4040051.

[4] R. Sinha , A. Debroy , N. Kumar , and H. Mondal , “Applicability of ChatGPT in Assisting to Solve Higher Order Problems in Pathology,” Cureus 15 (2023): e35237, 10.7759/cureus.35237. 36968864 PMC10033699

[5] K. J. Hewitt , I. C. Wiest , Z. I. Carrero , et al., “Large Language Models as a Diagnostic Support Tool in Neuropathology,” Journal of Pathology: Clinical Research 10 (2024): e70009, 10.1002/2056-4538.70009. 39505569 PMC11540532

[6] G. Broggi , M. Mazzucchelli , S. Salzano , et al., “The Emerging Role of Artificial Intelligence in Neuropathology: Where Are We and Where Do We Want to Go?,” Pathology, Research and Practice 263 (2024): 155671, 10.1016/j.prp.2024.155671. 39490225

[7] A. Esteva , B. Kuprel , R. A. Novoa , et al., “Dermatologist‐Level Classification of Skin Cancer With Deep Neural Networks,” Nature 542, no. 7639 (2017): 115–118, 10.1038/nature21056. 28117445 PMC8382232

[8] T. Hirosawa , R. Kawamura , Y. Harada , et al., “ChatGPT‐Generated Differential Diagnosis Lists for Complex Casederived Clinical Vignettes: Diagnostic Accuracy Evaluation,” JMIR Medical Informatics 11 (2023): e48808, 10.2196/48808. 37812468 PMC10594139

[9] M. L. Oon , N. L. Syn , C. L. Tan , K. B. Tan , and S. B. Ng , “Bridging Bytes and Biopsies: A Comparative Analysis of ChatGPT and Histopathologists in Pathology Diagnosis and Collaborative Potential,” Histopathology 84, no. 4 (2024): 601–613, 10.1111/his.15100. 38032062

[10] B. E. Bejnordi , M. Veta , P. J. Van Diest , et al., “Diagnostic Assessment of Deep Learning Algorithms for Detection of Lymph Node Metastases in Women With Breast Cancer,” JAMA: The Journal of the American Medical Association 318, no. 22 (2017): 2199–2210, 10.1001/jama.2017.14585. 29234806 PMC5820737

[11] D. Cirillo , S. Catuara‐Solarz , C. Morey , et al., “Sex and Gender Differences and Biases in Artificial Intelligence for Biomedicine and Healthcare,” Npj Digital Medicine 3, no. 1 (2020): 81, 10.1038/s41746-020-0288-5. 32529043 PMC7264169

[12] M. V. Heinz , S. Bhattacharya , B. Trudeau , et al., “Testing Domain Knowledge and Risk of Bias of a Large‐Scale General Artificial Intelligence Model in Mental Health. Digit,” Health 9 (2023): 9, 10.1177/20552076231170499. PMC10123874 37101589

[13] G. Campanella , M. G. Hanna , L. Geneslaw , et al., “Clinical‐Grade Computational Pathology Using Weakly Supervised Deep Learning on Whole Slide Images,” Nature Medicine 25, no. 8 (2019): 1301–1309, 10.1038/s41591-019-0508-1. PMC7418463 31308507

[14] L. Pantanowitz , G. M. Quiroga‐Garza , L. Bien , et al., “An Artificial Intelligence Algorithm for Prostate Cancer Diagnosis in Whole Slide Images of Core Needle Biopsies: A Blinded Clinical Validation and Deployment Study,” Lancet Digital Health 2, no. 8 (2020): e407–e416, 10.1016/S2589-7500(20)30159-X. 33328045

[15] G. Broggi , A. Maniaci , M. Lentini , et al., “Artificial Intelligence in Head and Neck Cancer Diagnosis: A Comprehensive Review With Emphasis on Radiomics, Histopathological, and Molecular Applications,” Cancers 16, no. 21 (2024): 3623, 10.3390/cancers16213623. 39518063 PMC11545333

[16] J. Y. Cheng , J. T. Abel , U. G. J. Balis , D. S. McClintock , and L. Pantanowitz , “Challenges in the Development, Deployment, and Regulation of Artificial Intelligence in Anatomic Pathology,” American Journal of Pathology 191, no. 10 (2021): 1684–1692, 10.1016/j.ajpath.2020.10.018. 33245914

[17] J. Lee , “Is Artificial Intelligence Better Than Human Clinicians in Predicting Patient Outcomes?,” Journal of Medical Internet Research 22, no. 8 (2020): e19918, 10.2196/19918. 32845249 PMC7481865

